# Effects of eight-week high-intensity interval training on some metabolic, hormonal and cardiovascular indices in women with PCOS: a randomized controlled trail

**DOI:** 10.1186/s13102-023-00653-z

**Published:** 2023-03-29

**Authors:** Somayeh Mohammadi, Amirabbas Monazzami, Solmaz alavimilani

**Affiliations:** 1 Department of Sport Physiology, Faculty of Sport Sciences, Razi University, Kermanshah, Iran; 2 Department of Obstetrics and Gynecology, Imam Reza Hospital, Kermanshah University of Medical Sciences, Kermanshah, Iran

**Keywords:** HIIT, HOMA-IR, Hormonal status, VAI, AIP, PCOS

## Abstract

**Background:**

Studies have revealed that high-intensity interval training (HIIT) has beneficial effect on hormonal, cardiovascular indices in women with polycystic ovary syndrome (PCOS). There, however, is still no comprehensive data on the type, intensity and duration of training for these women.

**Objective:**

The current study aimed to investigate the effects of high-intensity interval training (HIIT) on metabolic, hormonal and cardiovascular indices in women with PCOS compared to a control group.

**Methods:**

In a randomized controlled study, 28 patients (age: 23.8 ± 5.3 years, weight: 82.4 ± 9.7 kg, BMI: 30.33 ± 3.99 kg/m^2^) were divided into two groups including HIIT (n = 14) and the control (n = 14). The training protocol was performed with 100–110 maximum aerobic velocity (MAV), 4–6 sets, 4 laps, 3 sessions per week for eight weeks. Anthropometric indices, aerobic performance, insulin resistance and sensitivity, lipid profiles, testosterone, cortisol and hs-CRP were evaluated.

**Results:**

The HIIT intervention decreased BMI, waist to hip ratio (WHR), visceral fat, insulin, insulin resistance, low density lipoprotein (LDL), atherogenic index, cholesterol and cortisol (*P* < 0.05). All variables remained unchanged in the control group (*P* > 0.05). Except for VAI, FBG, HDL, TG and AIP, the rest of the variables in the training and control groups show a significant difference (*P* < 0.05).

**Conclusion:**

The results of the present study indicate that eight weeks of HIIT has beneficial effects on anthropometric, insulin sensitivity, fat profile, and inflammatory and cardiovascular indices in PCOS patients. It seems that the intensity of HIIT (100–110 MAV) is a determining factor in creating optimal adaptations in PCOS patients.

*Trail registration*: IRCT20130812014333N143. Registration date: 22/03/2020. URL: https://en.irct.ir/trial/46295.

## Introduction

Polycystic ovary syndrome (PCOS) is the most common endocrine disorder in women of reproductive age and is the leading cause of infertility and anovulation [1, 2]. According to the Rotterdam criteria, the prevalence of PCOS is estimated to affect up to 20% of the female population [3, 4]. PCOS is characterized by increased androgen production and decreased ovulation leading to clinical manifestations including acne, hirsutism, male pattern baldness, irregular menstrual cycles, and infertility [4–7]. In addition to concerns about fertility and hyperandrogenism, PCOS is considered a metabolic disorder, with an increased risk of developing insulin resistance (IR), hyperinsulinemia, dyslipidemia, and low-grade inflammation [8].

Moreover, dyslipidemia is the most common metabolic abnormality in PCOS in which the particle size of low-density lipoprotein (LDL) in PCOS women is excessive, and the particle size of high-density lipoprotein (HDL) is reduced. In addition, insulin resistance is associated with increased cholesterol levels and decreased HDL levels. Elevated levels of cholesterol, LDL, triglycerides, and low-grade inflammation have been identified as the associated factors in predicting CVD [9].

Measuring the risk of predictable factors, the atherogenic index of plasma (AIP) has also been proposed as a new indicator to predict the risk of cardiovascular disease (CVD) [10]. Furthermore, hs-CRP and homocysteine levels, out of the aforesaid risk factors, are strongly associated with insulin resistance and women with PCOS [11]. Homocysteine independently and strongly predicts the risk of stroke, heart attack, cardiovascular disease and sudden death in healthy individuals [12–16]. Elevated hs-CRP can also predict cardiovascular complications in both low-risk and high-risk populations. It has also been observed that people with low CRP levels are half as likely to have a heart attack [17].

Furthermore, women with PCOS experience more obesity; even thin women with PCOS often have higher levels of visceral fat [18–22]. More recently, visceral obesity has been defined as low-grade inflammation [21, 22]. Chronic low-grade inflammation, described in women with PCOS, is a potential link between hyperandrogenism, insulin resistance, or abdominal obesity and the long-term consequences of the syndrome [21, 22]. Accordingly, it has been revealed that the visceral adiposity index (VAI) can be used as a valid indicator to predict abdominal obesity and insulin resistance [23].

Inadequate changes in insulin also lead to the production of internal androgens, while insulin resistance leads to hyperinsulinemia, a decrease in sex hormone-binding globulin (SHGB) and an increase in free testosterone (FT) in the bloodstream. However, cortisol levels will increase in women with PCOS indicating hormonal changes and increased stress and inflammation in these women. Due to these conditions, most women with PCOS need long-term treatment and commonly available medications for PCOS are effective, although they have many side effects. For example, Metformin and Spironolactone can cause stomach upset, dizziness, nausea, and a metallic taste in the mouth. Spironolactone can also increase periods in women with PCOS. Therefore, the focus is on non-pharmacological treatment strategies including an active lifestyle and regular exercise. This issue pays more attention to non-pharmacological treatment strategies, including active lifestyle and regular exercise; some solutions that have been suggested as a priority [24]. Accordingly, Santos et al. [25] conducted a systematic review to identify and describe the effect of exercise on clinical outcomes in PCOS. Measured outcomes included cardiovascular risk factors (insulin resistance, blood lipid profiles, and weight) and reproductive measures include ovulation, menstruation, and fertility outcomes.

In a study by Almenning et al. [26], they performed a randomized controlled trial on the effect of HIIT and resistance training on metabolic, cardiovascular, and hormonal variables in women with PCOS. The researchers concluded that HIIT improved insulin resistance without weight loss in the women with PCOS. Body composition also improved significantly after high-intensity strength training. This experimental study revealed that exercising can improve cardiovascular parameters in women with PCOS without weight loss [26]. A body of evidence has demonstrated comparable or superior improvements in cardiometabolic fitness using HIIT in comparison to moderate endurance training. Therefore, according to studies, despite the potentially beneficial effects of exercise in women with PCOS, there is a gap between the type and intensity of exercises which is needed to improve the outcomes in this population. HIIT has a greater impact on body composition, fat profile, and insulin resistance than aerobic and resistance training. Due to the wide variety in HIIT program (intensity, duration and frequency), the effects of any type of HIIT program with a certain intensity, duration and frequency cannot be prescribed for other HIIT programs. Therefore, each type of HIIT program has its own characteristics and creates its own adaptations. As a result, the primary goal of this study is to determine whether HIIT can reduce insulin resistance and increase cardiorespiratory fitness. A secondary aim was to determine whether HIIT can reduce anthropometric, cardiovascular indices and improve hormone levels in women with PCOS. This article is taken from large research project, the first article of which was published in the international journal of fertility and sterility (Vol 16, No 4, October–December 2022).

## Material and methods

### Study design

**Fig. 1:**
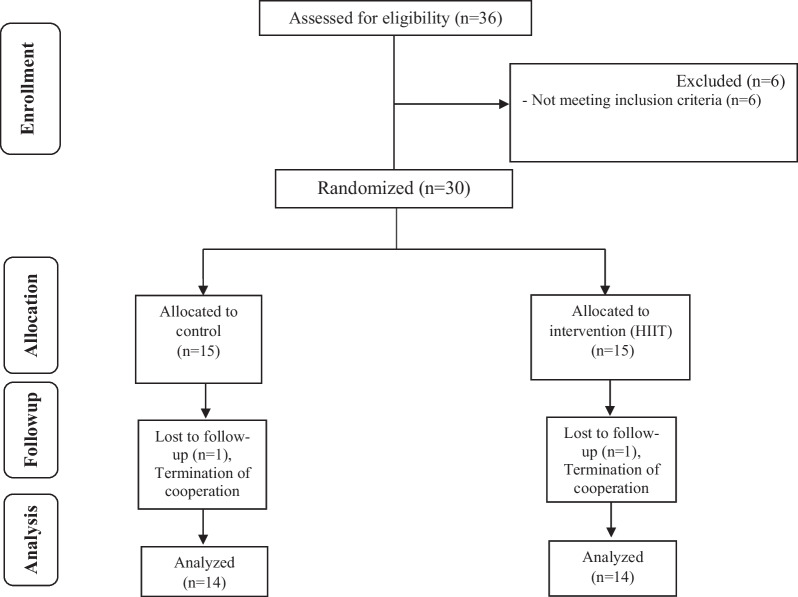
Diagram of the progress through the phases of the parallel randomized trial of two groups (enrolment, allocation, follow-up, and data analysis)

The present study was performed as a randomized parallel controlled trial from April 20th to October 20th, 2020 on patients who were referred to the gynecological clinic of Imam Reza Hospital, Kermanshah, Iran. Iran clinical studies registration center has confirmed this study with IRCT number: IRCT20130812014333N143 and registration date: 22-03-2020. URL: https://en.irct.ir/trial/46295. PCOS was diagnosed according to Rotterdam criteria [2]. Inclusion criteria were women aged 18–40 years and no history of exercise in the previous year. Exclusion criteria included following a weight loss diet, taking oral contraceptive pills, smoking and drinking alcohol and taking other effective drugs. Sample size was based on the formula to calculate the sample size with a significance level of less than 0.05, statistical power above 80%, and standard deviation ($\partial$) of the main research variables (insulin).This index is calculated based on the main variable of the study that has been measured in previous studies [26]. Target difference (D) was calculated based on the researcher's prediction from the research variable outcomes and finally, 28 patients were necessary (Formula 1) [26]. Accordingly, out of 36 women with PCOS, 30 were selected and randomly divided into two groups of high-intensity interval exercise (n = 14) and control (n = 14) (Fig. 1). Randomization was carried out from a computer-generated sequence (random number generator software), concealed in sequentially numbered, sealed, opaque envelopes (SNOSE method), and kept by the clinic technician of the two centers. Individuals understood all aspects of the study and provided informed consent. This study was approved by the Ethics Committee in Biomedical Research of the University of Medical Sciences, Kermanshah, Iran (the code number: IR.kums.REC.1398.1186).

1 $${\varvec{N}} = \user2{ }\frac{{4\user2{ }\left( \partial \right)^{2} \user2{ }\left( {{\varvec{Z}}_{{\user2{crit }}} + {\varvec{zp}}_{{{\varvec{wr}}}} } \right)^{2} }}{{{\varvec{D}}^{2} }} = \frac{{4\user2{ }\left( {4.4} \right)^{2} \user2{ }\left( {1.96 + 0.842} \right)^{2} }}{{5.5^{2} }} = \frac{{77.44 \times 7.85\user2{ }}}{30.25} = 20.096$$

### Outcomes of measurements

Anthropometric quantities were weighed via a professional technician at the Nasiri Diet Therapy Clinic at the beginning and the end of the trial. Height was measured by automatic stadiometer (Aneascale, Iran). Weight, body fat percent (FP), visceral adipose tissue (VAT) and BMI (body mass index) were determined by 3D body scanner (Anea 3D, Iran). To calculate the waist to hip ratio (WHR), waist circumference at the midpoint between the iliac crest and the lower rib margin and pelvic circumference (cm) at the point of maximum gluteal bulge were measured from the lateral view. Also, formula (2) was applied [23, 30–34] to calculate the intra-visceral fat index.

2 $${\text{VAI}}_{{{\text{women}}}} = \left( {\frac{WC}{{39.58 + 1.89 \times BMI}}} \right) \times \left( { \frac{TG}{{0.81}}} \right) \times \left( {\frac{1.52}{{HDL}}} \right)$$

Approximately 10 ml of peripheral blood was taken from PCOS patients to measure serum levels of research variables. Blood samples were taken from patients after 12–14 h of fasting in two stages, before and after eight weeks of training (48 h after the last training session). All biochemical variables were measured in MADAR laboratory. Blood samples after fasting were taken at the starting and the end point followed by immediate centrifuging (Hettich D-78532, Tuttlingen, Germany, 3500 rpm, 10 min) to isolate serum. At that point, the samples were kept at − 80 °C until next examinations. Then, enzymatic Kits (Pars Azmun, Tehran, Iran) were used to measure FPG and lipid profiles. Commercial ELISA kits were applied to examine hs-CRP (LDN, Germany), insulin, homocysteine, and cortisol (Monobind, California, USA). HOMA-IR and QUICKI were quantified based on the standard formula [23, 30–34]. The atherogenic index (AIP) was also calculated as Log (TG/HDL) [10]. To prevent the effect of circadian rhythm, blood sampling was performed at 8:00–9:00 in the morning. Subjects were asked to refrain from strenuous physical activity for 48 h before blood sampling [10, 23, 27–29].

### Training program

The training program was carried out for eight consecutive weeks with three sessions per week according to Oueroghi et al. [30–32]. HIIT sessions were performed in Torange club in the afternoon three days per week. The high-intensity interval training program included a warm-up for each session (including 15 min of standard warm-up), starting with a low-intensity run (50% of maximum aerobic speed) and then 3 repetitions of 30-s sprint running followed by 30 s of slow running and 5 min of dynamic stretching. HIIT program in the first week included interval running for 30 s with intensity of 100% maximum aerobic speed (MAV), 30 s of active recovery with 50% of maximum aerobic speed, 4 sets, 4 laps and 5 min of inactive recovery between each lap, 3 sessions per week for 8 consecutive weeks. The number of sets and laps for the HIIT program increased according to Table 3 for the following weeks (Table 1). All training sessions were supervised by an international coach. Multi-stage fitness tests (MSFT) were carried out to determine aerobic power on the treadmill. The speed of the subjects started from 8.5 km/h for one minute. In each stage, the speed increased by 0.5 km/h for each patient. Finally, the aerobic power was calculated using a formula (3).

3 $${\text{VO}}_{2\max } = 6\left( {{\text{measured}}\;{\text{speed}}} \right) - 22.4$$

**Table 1:** Eight weeks of high intensity interval training (HIIT) program

Training program	Weeks of training			
	First, second	Third, fourth	Fifth, sixth	Seventh, eighth
LAP	4	4	4	4
SET	4	6	6	6
Exercise/rest (sec)	30:30	30:30	30:30	30:30
Exercise/rest (intensity: percent)	100:50	100:50	100:50	100:50
Rest (min)	5	5	5	5

The patients in the control group were required not to refrain from additional exercise during the program [30–37].

### Statistical analysis

Descriptive statistical methods were applied to describe the mean and standard deviation of the data. The normality of the data distribution was estimated using the Shapiro–wilk test. Two-way analysis of variance with repeated measures was used to compare the means of the data in two groups (Delta, ∆). Moreover, Bonferroni post hoc test was used to compare the data changes in pre-test and post-test in each group. The effect size (ES) was calculated applying Cohen’s d; these calculations were based on Cohen’s classification of a small (0.2 < ES < 0.5), moderate (0.5 < ES < 0.8) and large (ES ≥ 0.8) effect size [27]. A *P*-value < 0.05 based on two-sided calculation was considered significant. Calculations were performed using SPSS software version 21.

## Results

As shown in Fig. 1, 36 women with PCOS participated in this study and 6 patients were excluded from the study based on the inclusion criteria. Finally, 30 people were randomly divided into two groups of control (N = 14) and HIIT exercise (N = 14). During the implementation of the training protocol, 2 people were excluded from the study and finally 28 people entered the final analysis. As shown in Table 2, there were no significant variances between participants considering age, height, BMI and weight at baseline.

**Table 2:** General characteristics of the participants

Variables	Control group	HIIT group	*P* value*
Age (y)	22.9 ± 5.3	24.2 ± 4.8	0.51
Height (cm)	162.5± 5.7	163.1 ± 5.0	0.78
Weight-baseline (kg)	84.2 ± 6.5	77.1 ± 12.4	0.70
BMI- baseline (kg/m^2^)	31.4 ± 2.6	29.5 ± 4.5	0.18

All values are means ± SD

*BMI*, Body mass index

*Obtained from independent t- test

**Fig. 2:**
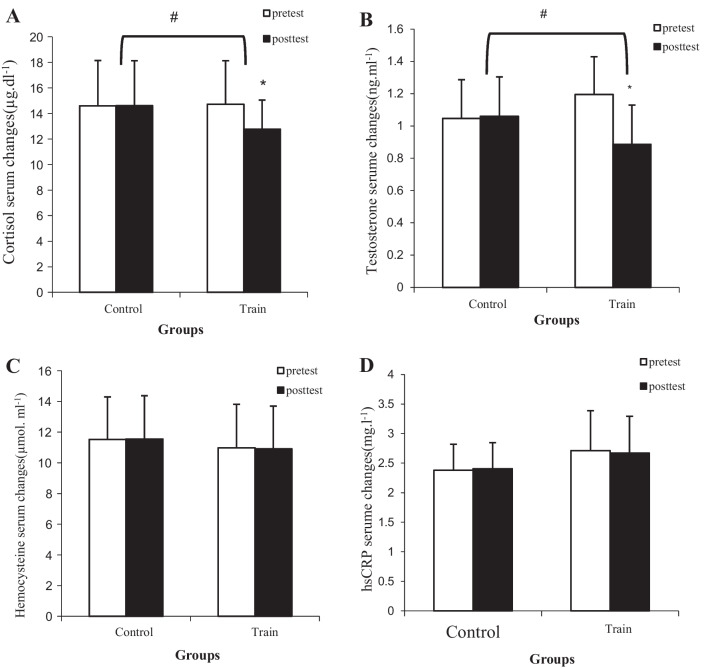
Changes in the Cortisol, testosterone, homocysteine and hsCRP serum levels of the studied groups (mean and standard deviation). **A** Changes in serum cortisol levels in research groups. **B** Changes in serum testosterone levels in research groups. **C** Changes in homocysteine serum levels in research groups. **D** Changes in high-sensitivity C-Reactive- Protein(hs-CRP) serum levels in research groups. ^a^Significant difference with pre-test (*P* < 0.05), ^b^Significant difference with the changes of the control group (∆) (*P* < 0.05)

There was no significant difference in any of the variables at baseline. After eight weeks of HIIT, the findings revealed that VAI (*P* = 0.19), glucose (*P* = 0.30), HDL (*P* = 0.70), TG (*P* = 0.08), AIP (*P* = 0.29), homocysteine (*P* = 0.21) and hs-CRP (*P* = 0.36) were not significantly different from the pre-test, but these changes were significant (*P* = 0.001) (Table 3, Fig. 2) in weight (*P* = 0.001), BMI (*P* = 0.001), fat percentage (*P* = 0.001), WHR (*P* = 0.001), VAT (*P* = 0.001), VO2max (*P* = 0.001), insulin (*P* = 0.001), HOMA-IR (*P* = 0.001), QUICKI (*P* = 0.001), LDL (*P* = 0.001), TC (*P* = 0.001) and TS/C ratio (*P* = 0.009) (Table 3, Fig. 2). The results also showed that after eight weeks of HIIT, anthropometric indices, lipid profile and insulin and HOMA-IR decreased significantly (*P* = 0.001), while aerobic performance (*P* = 0.001) increased significantly compared to the control group. The findings also revealed that despite the decrease in visceral fat indices (*P* = 0.25), glucose (*P* = 0.25), HDL (*P* = 0.33), TG (*P* = 0.33), homocysteine (*P* = 0.25) and hs-CRP (*P* = 0.17), were not significant compared to the control group (Fig. 2 and Table 3). In the variables of weight (*P* = 0.001), BMI (*P* = 0.001), fat percentage (*P* = 0.001), WHR (*P* = 0.001), VAT (*P* = 0.001), VO2max (*P* = 0.001), insulin (*P* = 0.001), HOMA-IR (*P* = 0.001), QUICKI(*P* = 0.001), LDL(*P* = 0.001), TC (*P* = 0.001), AIP(0.027) and TS/C ratio (*P* = 0.001), a significant difference was observed between the training and control groups (Table 3).

**Table 3:** Changes of glucose metabolism, lipid profile at the baseline and after eight weeks in patients with PCOS (mean and standard deviation)

Variables	Control group				HIIT group				*P* value^b^	*ƞ*
	Baseline	Week 8	Change	*P* value^a^	Baseline	Week 8	Change	*P* value^a^		
Weight (kg)	84.25 ± 6.8	84.39 ± 6.5	0.14 ± 0.56	0.46	77.1 ± 12.4	74.6 ± 12.5	2.50 ±0.85	0.001	0.001	0.78
BMI (kg/m^2^)	31.42 ± 2.6	31.50 ± 2.7	0.14 ± 1.02	*0.47*	29.52 ± 4.5	28.39 ± 4.47	1.13 ± 0.15	0.001	0.001	0.44
Body fat (percent)	29.79 ± 2.1	30.03 ± 2.4	0.24 ± 0.59	0.26	29.28 ± 2.3	27.04 ± 2.6	1.81 ± 0.95	0.001	0.001	0.64
WHR(m)	0.92 ± 0.03	0.93 ± 0.04	0.01 ± 0.019	0.06	0.91 ± 0.04	0.89 ± 0.04	0.02 ± 0.021	0.001	0.001	0.40
VAT (cm^2^)	118.5 ± 25	118.7 ± 25	0.17 ± 1.07	0.77	121.3 ± 16.7	118.1 ± 17.2	3.2 ± 2.94	0.001	0.001	0.38
VAI	6.88 ± 0.71	6.92 ± 0.59	0.03 ± 0.30	0.75	6.71 ± 1.24	6.55 ± 1.16	0.15 ± 0.54	0.19	0.25	0.049
VO2max (ml kg^−1^ min^−1^)	29.35 ± 1.7	29.14 ± 1.9	0.21 ± 0.57	0.31	30.7 ± 2.3	34.07 ± 2.5	3.35 ± 0.92	0.001	0.001	0.85
FPG (mg/dl)	98.2 ± 5.2	98.6 ± 4.2	0.42 ± 1.55	0.53	96.9 ± 5.5	96.2 ± 4.07	0.71 ± 3.29	0.30	0.25	0.05
Insulin (µU/ml)	10.85 ± 1.46	11.10 ± 1.48	0.25 ± 0.67	0.33	11.21 ± 1.50	8.96 ± 1.04	2.25 ± 1.17	0.001	0.001	0.64
HOMA-IR	2.63 ± 0.38	2.70 ± 0.38	0.07 ± 0.15	0.23	2.67 ± 0.34	2.13 ± 0.27	0.60 ± 0.25	0.001	0.001	0.67
QUICKI	0.33 ± 0.007	0.32 ± 0.007	0.001 ± 0.003	0.33	0.32 ± 0.006	0.34 ± 0.006	0.01 ± 0.006	0.001	0.001	0.64
LDL-C (mg/dl)	104.8 ± 9.4	105.2 ± 9.05	0.39 ± 1.96	0.50	104.5 ± 8.6	95.7 ± 7.5	8.71 ± 2.36	0.001	0.001	0.82
HDL-C (mg/dl)	37.5 ± 4.41	37.0 ± 4.48	0.57 ± 1.01	0.31	38.2 ± 5.15	38.5 ± 5.63	0.21 ± 2.77	0.70	0.33	0.03
Total-Chol (mg/dl)	170.0 ± 12.03	170.6 ± 12.11	0.64 ± 1.49	0.51	171.2 ± 15.47	164.07 ± 16.1	7.21 ± 4.90	0.001	0.001	0.55
Triglyceride (mg/dl)	148.6 ± 13.14	148.2 ± 12.81	0.42 ± 3.03	0.70	151.14 ± 8.52	149.14 ± 8.98	2.0 ± 5.9	0.08	0.33	0.03
AIP	0.59 ± 0.06	0.60 ± 0.05	0.005 ± 0.01	0.44	0.59 ± 0.06	0.59 ± 0.06	0.007 ± 0.035	0.29	0.2	0.06
TS/C ratio	0.079 ± 0.025	0.080 ± 0.024	0.001 ± 0.005	0.72	0.091 ± 0.037	0.081 ± 0.025	0.01 ± 0.018	0.009	0.03	0.16

*BMI*, Body mass index; *WHR*, Waist to hip ratio; *VAT*, Visceral adipose tissue; *VAI*, Visceral adiposity index; *VO2max*, Maximum oxygen uptake; *FPG*, Fasting plasma glucose; *HOMA-IR*, Homeostatic model assessment for insulin resistance; *QUICKI*, Quantitative insulin-sensitivity check index***;**** LDL-C*, Low density lipoprotein- cholesterol; *HDL*, High density lipoprotein- cholesterol; *AIP*, Atherogenic index of plasma; *TS/C ratio*, Testosterone to cortisol ratio

^a^Significant difference with pre-test (*P*< 0.05)

^b^Significant difference with the changes of the control group (∆) (*P* < 0.05)

*P* values calculated using two-way analysis of variance followed with Bonferroni's post-hoc test

Ƞ Effect size of training protocol

## Discussion

The present study is the first study to investigate the effects of HIIT (110–100% MAV, 4–6 sets and 4 laps) on anthropometric, lipid profile, insulin sensitivity and resistance, hormonal and inflammatory indices in PCOS patients. The results showed that, after eight weeks, HIIT reduced anthropometric indices, HOMA-IR, LDL, TC, androgenic and inflammatory biomarkers significantly and increased insulin sensitivity and aerobic function. It is also revealed that after eight weeks of HIIT, body weight, fat percentage, waist-to-hip ratio, and visceral fat decreased and VO2max increased. Moreover, the findings indicated that despite the decrease in visceral fat index, it was not significant. Supporting the results, Hutchison et al. [38] showed that HIIT reduced visceral fat. In a crossover study, Roessler et al. [39] demonstrated that HIIT reduced weight and waist circumference and increased VO2max. In a randomized controlled study, Almenning et al. [26] revealed that HIIT and resistance trainings reduced body composition and increased VO2max. They reported that VO2max was greater in the HIIT group. Contrary to these findings, Lionett et al. [40] reported that HIIT did not improve fat oxidation, and this lack of change indicate metabolic inflexibility in PCOS women. Maillard et al. [41] and Santos et al. [25] in two separate meta-analysis studies showed that HIIT can be used as a time-efficient strategy to reduce visceral fat and improve body composition [41, 42]. Discrepancies could be related to differences in subject characteristics (ethnicity, smoking, alcohol consumption, dietary intake, and physical fitness), training intensity, type and duration. The mechanisms that increase VO2max that go beyond the present study, although, it may be due to improved heart output and O2 heart rate. In a study, Dussin et al. [43] they showed that after two months of HIIT, cardiac output and stroke volume increased in inactive individuals. Perry et al. [44] also reported that six weeks of HIIT increased the mitochondrial content of several proteins (citrate synthase, malate dehydrogenase, pyruvate dehydrogenase) by 18–29 percent. This increase in fat oxidation occurs after HIIT due to the need for energy to neutralize protons and increase the regeneration of glycogen and phosphocreatine [44].

Fat accumulation, especially in peripheral adipose tissue, is the most important factor causing metabolic diseases such as PCOS. Visceral adipose tissue participates in the development of chronic inflammation, insulin resistance and cardiovascular disease through the secretion of proinflammatory agents such as cytokines and hormones. There is ample evidence that HIIT reduces abdominal and visceral adipose tissue by increasing fat metabolism and reducing the risk of accumulated fat compared to low- and moderate-intensity continuous aerobic exercise methods. Mechanisms that increase periodicity of fat metabolism include increasing the capacity and content of mitochondria and the activity of catecholamines, which break down visceral fat which is due to its beta-adrenergic receptors relative to subcutaneous fat leading to an increase in energy expenditure and a decrease in appetite after exercise. Therefore, many studies have reported that HIIT can be used more attractively than continuous exercises due to time efficiency and adherence to training [39, 40].

The results also indicated that HIIT has been successful in reducing insulin resistance. These changes are due to decreased plasma insulin levels, not glucose changes. Increased insulin increases the secretion of androgens from the ovaries, which inhibits the secretion of SHBG from liver and worsens the condition in these patients. Therefore, reducing insulin levels and increasing insulin sensitivity can improve the quality of life in these patients. Supporting the current findings, Almenning et al. [26] showed that insulin resistance decreased after 10 weeks of HIIT, but Lionett et al. [40] revealed that 16 weeks of HIIT had no effect on insulin resistance. Hutchison et al. [38] reported that 12 weeks of high-intensity aerobic exercise reduced insulin resistance in these patients. Brown et al. [45] also found reported that performing 24 weeks of moderate-intensity aerobic exercise had no significant effect on insulin sensitivity and resistance in women with PCOS. Different results can be due to the duration, intensity, the number of training sessions and the type of training used [45]. Furthermore, increased exercise may improve insulin sensitivity through increased muscle mass, as muscle contractions stimulate glucose uptake in the absence of insulin through increased glut-4 expression (Kristen Farrell, M.S.2010).

Moreover, it was suggested that interval exercise may affect the lipid profile. Compared to the control group, cholesterol and LDL levels decreased significantly, but despite the decrease in triglyceride levels and low HDL, these changes were not significant. In supporting our results, Sprung et al. [46] evaluated the effects of 12 weeks of moderate-intensity aerobic exercise on vascular endothelial function in women with PCOS. The results showed that despite the lack of weight loss in these patients, LDL and cholesterol levels significantly reduced, while triglyceride HDL levels did not significantly change. The main activator of lipolysis during exercise is sympathoadrenal system. The effect of beta-adrenergic is based on sympathetic nervous system or epinephrine simulation. Epinephrine is considered as the main activator of the sensitive lipase to hormones. During HIIT training, an increase in LPL enzyme activity and an increase in the area of capillary capacity led to refining the rate of VLDL and increasing cholesterol. Endothelial function is also improved, so the risk of cardiovascular disease is reduced [46]. The lack of changes in triglycerides and HDL indicates why the visceral fat index did not change significantly. However, the results of atherogenic index showed that there is a significant difference between exercise and control groups revealing the fact that HIIT may reduce the risk of cardiovascular disease [10, 47].

The results showed that despite the decrease in homocysteine and hs-CRP levels in HIIT group, these changes were not significant. Previous studies by Boshku et al. [48] have shown that hyperhemocysteinemia and increased hs-CRP can be used as the important factors in assessing the risk of cardiovascular disease in PCOS patients. These findings suggest that increased body weight and central fat are major basis of metabolic aberrations associated with CVD in PCOS, while hs- CRP is a marker indicating the existence of low-grade chronic inflammation and increased CVD risk [48]. Almenning et al. [26] showed that after 10 weeks of HIIT, despite the decrease in homocysteine and CRP levels, these differences were not significant in comparison to the control group, which is consistent with the findings of our study [26]. However, Miranda-Furtado et al. [49] revealed that homocysteine levels did not decrease after 4 months of resistance training in PCOS women. In contrast, Randeva et al. [50] demonstrated that 6 months of aerobic exercise was able to reduce homocysteine levels in these patients. The mechanisms by which exercise reduces homocysteine levels and subsequently reduces the progression of atherosclerosis are beyond the scope of this study, but it appears that increasing homocysteine reduces the availability of nitric oxide, thereby reducing vascular flexibility, and exercise by reducing homocysteine levels; they increase the availability of nitric oxide and shear stress of vascular vessels, thereby improving endothelial function [50].

The results of the present study showed that the ratio of testosterone to cortisol in the exercise group decreased significantly due to a significant decrease in testosterone and cortisol compared to the control group. Some studies have indicated that a combination of aerobic and resistance training reduces testosterone levels. Also, in the study of Samadi et al. [51], it was shown that 12 weeks of HIIT in water reduces testosterone levels. In the study by Kogure et al. [52], it was indicated that 4-month of resistance training also reduces testosterone levels. However, in a study by Almenning et al. [26], it was concluded that 10 weeks of HIIT did not change testosterone levels. Overall, the results of the present studies revealed that aerobic, resistance and HIIT increase muscle growth, especially glycolytic fibers to increase blood testosterone removal and improved androgen levels in PCOS patients. However, in a study by Tsilchorozidou et al., it was revealed cortisol levels increase in PCOS patients. The researchers also found that elevated insulin levels activated the enzymes 5α-R, 11Β-HSD1 and 20αβ-HSD, and that the activity of these enzymes disrupted steroid hormones. Therefore, exercise helps regulate the activity of this hormone by reducing the activity of these enzymes and reducing insulin levels and increasing insulin sensitivity [29]. However, research in this area is limited, and future studies could reveal some beneficial aspects of exercise training on levels of this hormone. The use of HIIT program with intensity of 100–110% of MAV for eight weeks is a strength of this study. The small sample size due to the lack of available subjects was one of the limitations of this study. Therefore, we suggest further similar studies with larger sample sizes. The training period was also another limitation that may have affected the results. Some other factors such as dietary intake and energy expenditure that may have affected the body composition and lipid profile were not controlled [53, 54].

## Conclusion

The findings from the current study revealed that eight weeks of HIIT had beneficial effects on anthropometric, aerobic function, insulin resistance and sensitivity, lipid profile, and inflammatory and cardiovascular indices in PCOS patients. These findings suggest that HIIT (100–110 MAV) may improve the cardio-metabolic profile in the PCOS patients. Further studies on the type, volume, intensity, frequency, and duration of HIIT are needed to obtain comprehensive data on HIIT protocols for these patients.

## Data Availability

All data generated or analyzed during this study are included in this published article.

## Abbreviations

PCOS: Polycystic ovary syndrome
HIIT: High intensity interval training
BMI: Body mass index
WHR: Waist to hip ratio
VAT: Visceral adipose tissue
FPG: Fasting plasma glucose
HOMA-IR: Homeostasis model of assessment-insulin resistance
QUICKI: Quantitative insulin sensitivity check index
IR: Insulin resistance
LDL-C: Low-density lipoprotein-cholesterol
HDL-C: High-density lipoprotein-cholesterol
AIP: Atherogenic index of plasma
TS/C ratio: Testosterone to cortisol ratio
hs-CRP: High sensitive-C reactive protein
T2BM: Type2 diabetes mellitus
CVD: Cardiovascular disease
CHD: Coronary heart disease
1RM: One repetition maximum
WHR: Waist to hip ratio
Vo2max: Maximal oxygen capacity
MAV: Maximum aerobic velocity
